# Could a remarkable decrease in leptin and insulin levels from colostrum to mature milk contribute to early growth catch-up of SGA infants?

**DOI:** 10.1186/s12884-017-1593-0

**Published:** 2017-12-06

**Authors:** Marina Nunes, Clécio Homrich da Silva, Vera Lucia Bosa, Juliana Rombaldi Bernardi, Isabel Cristina Ribas Werlang, Marcelo Zubaran Goldani, NESCA Group, Amanda Ferreira, Fabiana Copes, Mariana Lopes de Brito, Monique Cabral Hahn, Salete de Matos, Sara Brunetto, Tanara Vogel Pinheiro, Thamíris Medeiros

**Affiliations:** 1 Laboratório de Pediatria Translacional / Núcleo de Estudos em Saúde da Criança e do Adolescente (NESCA) – Hospital de Clínicas de Porto Alegre, Porto Alegre/RS, Brazil; 2 Programa de Pós-Graduação em Saúde da Criança e do Adolescente – Faculdade de Medicina, Universidade Federal do Rio Grande do Sul, Porto Alegre/RS, Brazil; 3 Departamento de Pediatria - Faculdade de Medicina, Universidade Federal do Rio Grande do Sul, Porto Alegre/RS, Brazil

**Keywords:** Breast milk, Leptin, Insulin, Adiponectin, Maternal body mass index, Small-for-gestational-age

## Abstract

**Background:**

Breast milk is known to contain many bioactive hormones and peptides, which can influence infant growth and development. In this context, the purpose of this study was to evaluate the influence of different clinical pregnancy conditions on hormone concentrations in colostrum and mature breast milk.

**Methods:**

An observational study was performed with mother-newborn pairs divided into five groups according to maternal clinical background: diabetes (12), hypertension (5), smoking (19), intrauterine growth restriction of unknown causes with small-for-gestational-age newborns at delivery (12), and controls (21). Socioeconomic data, anthropometric measurements and breast milk samples were collected between the first 24 and 48 h and 30 days postpartum. Leptin, adiponectin, and insulin levels in breast milk were measured by immunoassays.

**Results:**

A significant decrease in leptin (*p* = 0.050) and insulin (*p* = 0.012) levels from colostrum to mature breast milk in mothers of small-for-gestational-age infants was observed. Maternal body mass index was correlated with both leptin and insulin, but not with adiponectin. Insulin levels were negatively correlated to infant weight gain from birth to one month (*p* = 0.050). In addition, catch-up growth was verified for small-for-gestational-age infants throughout the first month of life.

**Conclusions:**

This study suggests that a remarkable decrease in leptin and insulin levels in mature milk of mothers of small-for-gestational-age newborns may be involved in the rapid weight gain of these newborns. The physiological and external mechanisms by which these significant decreases and rapid weight gains occur in this group remain to be elucidated.

## Background

Breast milk contains bioactive components, which play an important biological role for the newborn. Leptin, insulin and adiponectin are some of the hormones that possess important functions in energy balance regulation, food intake, and child body composition [1–3]. Leptin is an anorexigenic hormone that acts on neuronal cells of the hypothalamus [4]. It is produced by adipocytes, the human placenta and mammary glands [5–7]. Insulin displays anorexigenic effects similar to leptin [8]. It captures glucose for several tissues after food intake [9], and seems to interfere on enteral hormones slowing gastric emptying and promoting a sensation of satiety [10]. Adiponectin is secreted by adipose tissue and stimulates food intake and decreases energy expenditure, the inverse action of leptin and insulin [11, 12]. This hormone acts on glucose and lipid metabolisms, improving insulin sensitization and fatty acid oxidation, while also inhibiting hepatic glucose production [13, 14].

It is known that breast milk composition can be influenced by the maternal clinical background [11, 12], in which the body fat deposits are proportionally associated to leptin, adiponectin and insulin levels. Positive correlations have been demonstrated between maternal body mass index (BMI) and hormone concentrations in breast milk [12, 15–20], and between maternal BMI and serum leptin concentrations in breastfed infants [21].

Insulin quantification in the breast milk of diabetic mothers has been described by Whitmore et al. [22], who observed no differences in insulin levels over 24 h or in relation to diabetes type. Breast milk components can be modified by smoking, which induces variations in the concentration of certain cytokines [23–25]. However, no such changes have been observed for leptin or other hormones [24, 26]. Liu et al., assessed adiponectin concentrations in the breast milk of mothers presenting preeclampsia and observed increases when compared to controls [27].

In this context, the aim of the present study was to evaluate the influence of different clinical conditions during pregnancy (diabetes, hypertension, smoking and idiopathic intrauterine growth restriction) on hormone concentrations in colostrum and mature breast milk and their associations with the early development of the newborns.

## Methods

The present prospective and observational study was part of a larger project, named *Impact of Perinatal Different Intrauterine Environments on Child Growth and Development in the First Six Months of Life (IVAPSA Birth Cohort Study)*. The research protocol has been previously published [28]. The recruitment for the IVAPSA study was carried out in public hospitals - the Hospital de Clínicas de Porto Alegre and the Grupo Hospitalar Conceição (Hospital Fêmina and Hospital Conceição). Both are similar in maternal and child care and are located in Porto Alegre, Rio Grande do Sul, Brazil. Mothers were recruited at 24 to 48 h postpartum and were divided into five groups according to maternal clinical background and pregnancy outcome: (I) gestational diabetes treated only by diet (GDM) (II) gestational hypertension without intrauterine growth restriction (HTN). These maternal clinical backgrounds (diabetes and hypertension) were previously diagnosed by prenatal physicians; (III) smoking (SMS) – mothers who smoked at any time during the pregnancy. Smoking mothers were selected by asking whether they had smoked during their pregnancy (1 cigarette or more); (IV) idiopathic intrauterine growth restriction with small-for-gestational-age (SGA) newborn at delivery – birth weight below 5th percentile [29]; and (V) control (CTL) – healthy mothers without diabetes or hypertension, who did not smoke during their pregnancy and who did not deliver an SGA baby for unknown causes. Exclusion criteria for all groups were HIV-positive mother status, preterm delivery (< 37 gestational weeks), newborn twin, newborn presenting malformations at birth or newborn requiring any hospitalization after delivery.

Two observations were performed, the first one between 24 to 48 h after delivery (PP) at the hospital, and the second at one month postpartum (1 M). Two-hundred and fourteen mother-newborn pairs were recruited (GDM – 25; HTN – 23; SMS – 58; SGA – 25; CTL – 83). The samples comprised mothers whose colostrum and mature breast milk were available. Many of the elected mothers did not provide breast milk, either during the postpartum period, at 1-month postpartum, or at any timeframe at all. In some cases, the sample amount was insufficient for all the conducted analyses. Information on birth, prenatal, social, economic, family, and health data were obtained during interviews, using a structured questionnaire developed by the investigators. Anthropometric measurements were also obtained: height and weight were measured using a portable stadiometer (AlturaExata®) and balance (Marte®), respectively. Infant weight gain was expressed from Z-scores for age according to the World Health Organization growth curves [30].

Breast milk (1–5 mL) was collected according to the availability of each mother in the first 24 and 48 h after delivery and 30 days postpartum. No control if mothers had breastfed their infants recently was performed, precluding the determination of whether the collected sample was hindmilk or foremilk. Previously published results have shown no significant difference in leptin [1, 22, 31–33] and insulin [22] levels between breast milk samples obtained from the initial and terminal phases of suckling. Maternal fasting status was also not controlled. After milk expression into sterile flasks by manual milking, the samples were immediately fractionated into labeled 1.5-mL tubes and stored at −80 °C until analysis. Prior to the assays, the milk samples (colostrum and mature milk) were thawed and centrifuged at 15,000 rpm at 4 °C for 30 min to isolate fat in order to avoid its interference on the performed measurements. The isolated fat was discarded. No protease inhibitor was added.

Leptin, adiponectin and insulin were quantified in duplicate using commercially available Enzyme Linked Immuno Sorbent Assay (ELISA) kits (Millipore®) according to the manufacturer’s instructions.

Statistical analyses were performed using the Statistical Package for the Social Sciences software– version 18.0 (SPSS Inc.). The descriptive analyses were performed according to the parametric or nonparametric distribution of data, as identified by the Kolmogorov–Smirnov test. The chi-square test was used to verify sample homogeneity between groups, as was the Kruskal–Wallis test for continuous data. The Wilcoxon test was used for paired nonparametric data (to evaluate differences in breast milk hormone concentrations from colostrum to mature milk), and the differences between groups were assessed by the medians of the Kruskal-Wallis test applying the Games–Howell post-hoc test. An analysis of variance (ANOVA) test applying Tukey’s post-hoc test was used to compare the parametric variables of maternal BMI between groups. Spearman coefficients were used to assess potential nonparametric correlations between breast milk hormone levels and maternal BMI. A significance level of 5% (*p* ≤ 0.05) and 95% confidence intervals were considered.

## Results

Sixty-nine mother–child pairs were distributed across the five groups (12 GDM, 5 HTN, 19 SMS, 12 SGA, and 21 CTL). Of the mothers who provided breast milk in the first moment (colostrum), 9 (3 DM, 2 HTN, 1 SGA and 3 CTL) did not provide the second sample (mature milk). There were no significant differences in socioeconomic variables, as displayed in Table 1. Mothers of SGA newborns presented lower BMI values in all measurements.

**Table 1:** Socioeconomic and maternal characteristics in each group

	GDM (12)	HTN (5)	SMS (19)	SGA (12)	CTL (21)	*p*
Race, n (%)						
White	6 (50.0)	5 (100)	11 (57.9)	3 (25.0)	12 (57.1)	0.720
Marital situation, n (%)						
With a partner	11 (91.7)	4 (80.0)	16 (84.2)	10 (83.3)	16 (76.2)	0.853
Education Level, n (%)						
≤ 8 years	2 (16.7)	2 (40.0)	8 (44.4)	4 (33.3)	10 (52.6)	
9–11 years	10 (83.3)	3 (60.0)	10 (65.6)	8 (66.7)	9 (47.4)	0.358
Social Class^a^, n (%)						
B	2 (18.2)	3 (60.0)	4 (21.1)	3 (25.0)	7 (33.3)	
C	9 (81.8)	1 (20.0)	14 (73.7)	7 (58.3)	10 (47.6)	0.296
D	0 (0)	1 (20.0)	1 (5.3)	2 (16.7)	4 (19.0)	
Age (years), x (SD)	26.64 (5.75)	27.81 (11.22)	26.28 (5.96)	22.35 (5.64)	27.54 (7.49)	0.303
Mode of delivery, n (%)						
Cesarean	5 (41.7)	3 (60.0)	5 (26.3)	2 (16.7)	5 (23.8)	0.343
Maternal BMI (Kg/m^2^), x (SD)						
Pre-gestational	27.38 (5.72)**	26.82 (2.86)	23.90 (4.83)	21.09 (3.46)**	25.07 (5.73)	0.037*
At birth	31.86 (6.14)**	32.31 (4.06)**	30.24 (5,30)**	24.66 (2.77)**	30.95 (5.05)**	0.004*
At 1 month post-natal	28.26 (6.42)	29.57 (2.69)	26.62 (4.61)	22.32 (2.30)**	29.70 (5.57)**	0.020*

There were no statistically significant differences across groups in socioeconomic characteristics (p > 0.05 for chi-square test [categorical data] and Kruskal–Wallis test [continuous data]). Maternal BMI data: *p ≤ 0.05, ANOVA test between groups; **p ≤ 0.05, Tukey post-hoc test. ^*a*^ *Socioeconomic class assigned as per the Brazilian Economic Classification Criterion. SD: Standard Deviation; BMI: Body Mass Index; GDM: Gestational Diabetes Mellitus; HTN: Gestational Hypertension; SMS: Smoking; SGA: Small-for-gestational-age; CTL: Control*

All infants were still being breastfed during the second milk sampling of which forty-one were exclusively breastfeeding. Nineteen mothers offered other foods (solid, semi-solid or liquids) beyond breast milk and no differences in concurrent neonatal feeds and hormone concentration were observed (data not shown).

A significant difference between adiponectin concentrations of the HTN and CTL groups for colostrum (*p* = 0.015) was observed. Leptin and insulin concentrations decreased in all groups over time. These decreases were significant in mothers with SGA infants (*p* = 0.050), leading to the lowest leptin values of all groups at one month (*p* = 0.031) (Table 2).

**Table 2:** Breast Milk leptin, adiponectin and insulin concentrations at colostrum and mature milk according to groups

	GDM Median (25-75th)	HTN Median (25-75th)	SMS Median (25-75th)	SGA Median (25-75th)	CTL Median (25-75th)	*p*
Leptin, C (ng/mL)	0.668 (0.447–1.305)	0.970 (0.522–1.065)	0.599 (0.349–0.998)	0.641 (0.357–0.809)	0.813 (0.416–1.265)	0.715
Leptin, MM (ng/mL)	0.460 (0.450–0.702)	0.493 (0.485–0.591)	0.544 (0.443–0.800)	0.377 (0.357–0.412)***	0.715 (0.485–0.902)***	0.031**
p	1.000	0.655	0.753	0.050*	0.234	
Adiponectin, C (ng/mL)	10.230 (5.630–22.650)	20.880 (16.010–24.062)***	13.110 (10.090–18.040)	14.370 (8.535–21.465)	8.790 (6.902–11.352)***	0.015**
Adiponectin, MM (ng/mL)	12.430 (6.900–14.870)	14.710 (11.310–14.850)	10.190 (8.350–11.670)	9.990 (5.165–19.208)	9.870 (6.330–11.495)	0.754
p	0.109	0.180	0.112	0.123	0.959	
Insulin, C (μU/mL)	49.370 (25.700–176.540)	116.040 (66.315–154.325)	46.300 (26.960–121.950)	60.485 (18.322–103.597)	55.035 (11.567–162.642)	1.000
Insulin, MM (μU/mL)	22.830 (16.330–60.430)	26.000 (19.560–33.770)	22.800 (16.075–45.980)	16.665 (12.172–22.615)	22.030 (13.295–32.205)	0.330
p	0.273	0.180	0.084	0.012*	0.041*	

*p ≤ 0.05, Wilcoxon test for paired data; **p ≤ 0.05, Kruskal–Wallis test between groups; ***p ≤ 0.05, Games–Howell post-hoc test. (C): colostrum; (MM): mature milk; GDM: Gestational Diabetes Mellitus; HTN: Gestational Hypertension; SMS: Smoking; SGA: Small-for-gestational-age; CTL: Control

Leptin concentrations in colostrum and mature breast milk were correlated with pre-gestational maternal BMI, as well as at delivery and at one month post-natal, whereas insulin presented a similar correlation for mature milk samples. Adiponectin showed no correlation with maternal BMI during any of the evaluated time-frames (Table 3).

**Table 3:** Correlation of breast milk hormones with maternal BMI

	*n*	Correlation Coefficient	*p*
Pre-gestational BMI			
Leptin C	51	0.357	0.010*
Leptin MM	48	0.752	<0.001*
Adiponectin C	54	−0.038	0.787
Adiponectin MM	51	0.093	0.514
Insulin C	54	0.102	0.464
Insulin MM	52	0.330	0.017*
At birth BMI			
Leptin C	53	0.375	0.006*
Leptin MM	51	0.760	<0.001*
Adiponectin C	55	−0.157	0.253
Adiponectin MM	54	0.019	0.893
Insulin C	56	0.080	0.556
Insulin MM	55	0.359	0.007*
1 month post-natal BMI			
Leptin C	45	0.374	0.011*
Leptin MM	50	0.814	<0.001*
Adiponectin C	48	−0.193	0.189
Adiponectin MM	53	−0.026	0.851
Insulin C	47	0.087	0.561
Insulin MM	54	0.417	0.002*

*p ≤ 0.05, Spearman’s test. C: colostrum; MM: mature milk. BMI:body mass index

**Fig. 1:**
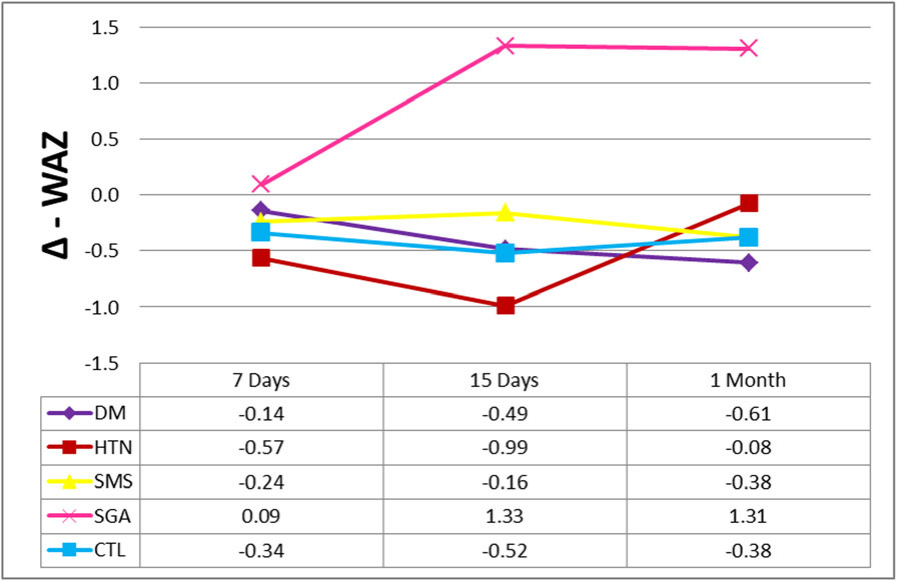
Differences in Weight-for-Age Z-scores (Δ – WAZ) in relation to birth weight in each group

Insulin in mature milk was negatively correlated to infant weight gain at one month (*p* = 0.050) (Table 4). A catch-up growth was observed in the SGA group at one month, as displayed in Fig. 1.

**Table 4:** Correlation of breast milk hormone concentrations with infant weight gain

	*n*	Correlation coefficient	*p*
Weight gain at 1 month			
Leptin, C	45	0.039	0.800
Leptin, MM	50	−0.264	0.064
Adiponectin, C	48	−0.112	0.450
Adiponectin, MM	53	−0.211	0.129
Insulin, C	47	−0.055	0.715
Insulin, MM	54	−0.268	0.050*

*p ≤ 0.05, Spearman’s test. C: colostrum; MM: mature milk

## Discussion

The evaluation of socioeconomic and maternal factors such as race, marital status, age, education level and social class showed a homogeneous sample, with similar characteristics despite the influence of different maternal gestational clinical conditions. Mothers of SGA infants presumably contributed through their lower BMI to restriction mechanisms of their offspring, although other factors may be involved, such as caloric restrictions or no apparent pathological conditions [34–36].

Results indicate no differences in insulin and leptin concentrations in colostrum regardless of the influence of the maternal gestational clinical condition, as previously demonstrated for diabetic [22] and smoking mothers [23, 26]. On the other hand, increased adiponectin levels have been observed for hypertensive mothers in comparison to controls, as described by Liu et al. [27]. Higher adiponectin serum and leptin levels in preeclampsia mothers have also been described by other authors [37, 38].

Decreases in hormone levels were observed during the first month of breastfeeding, in accordance to other studies [12, 17, 39–41]. Interestingly, breast milk from mothers of SGA infants exhibited a remarkable decrease in leptin and insulin levels at one month compared to other groups. Leptin and insulin breast milk levels correlate with their serum counterparts, which are proportional to maternal fat tissue [16, 19, 42]. Thus, these significant decreases can be related, at least in part, to the lower BMI of SGA mothers. A recent study demonstrated that the frequency and duration of breastfeeding presented a positive correlation with protein concentrations in breastmilk [43]. However, some authors have demonstrated that leptin levels are not modified comparing before and after suckling [1, 22, 31–33].

Maternal leptin and insulin serum concentrations during pregnancy increase concomitantly with maternal BMI [16, 44, 45], whereas adiponectin levels decrease [46]. Several studies have demonstrated a correlation between maternal BMI and satiety hormones in breast milk and serum of both mothers and neonates at delivery, as well as a broad variation in hormone levels [16–21, 42]. Herein, leptin concentrations in colostrum and mature milk positively correlated to maternal BMI before pregnancy, at delivery and at one month postpartum. Studies have demonstrated the same correlation for both colostrum [14, 17] and mature milk [6, 15, 17, 19, 47] with pre-gestational BMI, as well as maternal BMI at delivery [14] and at one month postpartum [1].

Maternal obesity has been associated with several adverse effects in children, such as metabolic, neurological and cardiovascular disorders later in life [48–51]. Increased leptin levels delivered to the fetus during pregnancy and breastfeeding may lead to predisposition for leptin resistance, impairing the ability of leptin to regulate appetite [52–55]. On the other hand, intrauterine growth restriction (IUGR) may also lead to leptin resistance [55–58]. Wattez et al. [59] have demonstrated leptin/insulin resistance in offspring from both undernourished dams and dams fed an obesogenic diet. The timing of neonatal leptin increases influenced by fetal nutrition contributes to the development of obesity in later life [58]. This reinforces the importance of maternal weight control before and during pregnancy in order to avoid adverse outcomes to the newborn.

Insulin levels in mature milk were also positively correlated to maternal BMI at each measurement period. However, no correlation was verified for colostrum samples. Few studies linking insulin concentrations in breast milk with maternal BMI are available. Ley and collaborators reported similar findings regarding pre-gestational BMI [12], while other researchers found no correlation between insulin levels and maternal BMI [15, 60]. In the present study, no correlation was observed between adiponectin concentrations and maternal BMI in any of the evaluated timeframes, in accordance to the literature [6, 7, 12, 41, 47, 61].

Insulin levels were negatively correlated with infant weight gain at one month of age, in accordance to its role as an energy balance, food intake and body composition regulator. In addition, high exposure to insulin during the lactation period has been associated with lower infant weight and lower lean mass at one month [15]. Insulin signaling pathways are involved in the development of the infant gastrointestinal system [10] and could act in the arcuate nucleus of the hypothalamus, affecting satiety and appetite control [8, 9]. Thus, it may influence postnatal growth.

The present study also demonstrates a catch-up growth of SGA infants through one month, which was not been observed for any other group. Rapid weight gain in both infancy and early childhood is a risk factor for adult adiposity and obesity [62]. In addition, rapid infant weight gain has also been associated with increased risk of being overweight at 4 years of age, independently of the potential confounders [62] and other unfavorable outcomes [56, 63–66]. Although early catch-up growth appears to be beneficial for the child, the Latin American SGA Consensus Guidelines recommend that children born SGA should not be allowed to gain weight too rapidly or excessively, probably in an effort to circumvent the development of metabolic disturbances [67].

The strengths of this study are the completeness of the collected maternal and neonatal data and the comparison of the impact of different maternal gestational clinical conditions on hormone concentrations in breast milk in a homogeneous sample. Some limitations are noteworthy, such as the lack of time control for the breast milk sampling or maternal fasting status, despite the fact that some studies have found no influence of these factors on hormone measurements [1, 22, 31–33]. Furthermore, the rigorous methodology for inclusion in the sample groups restricted the sample size.

## Conclusions

The results obtained herein demonstrate different patterns of hormone concentrations in breast milk from mothers of SGA newborns. The significant decreases in leptin and insulin concentrations after birth may be involved in weight regain as usual in SGA newborns. Interestingly, these changes occurred after birth, suggesting that post-natal and even external mediating factors could contribute to this outcome. Further investigations are required to clarify these findings.

## Abbreviations

1 M: 1 month postpartum
ANOVA: Analysis of variance
BMI: Body mass index
CTL: Control
ELISA: Enzyme Linked Immuno Sorbent Assay
GDM: Gestational Diabetes Mellitus
HTN: Gestational Hypertension
PP: Postpartum
SGA: Small-for-gestational-age
SMS: Smoking
